# AI-assisted vocal emotion analysis in forensic interview with children: an exploratory study

**DOI:** 10.3389/fpsyg.2026.1818889

**Published:** 2026-06-05

**Authors:** Yoonseo Lee, Seho Maeng, Yujin Kim, Mirae Hong, Seohyun Kang, Jinyoung Park, Haeun Chu, Jooyoung Lee

**Affiliations:** Dongduk Women's University, Seoul, Republic of Korea

**Keywords:** AI-assisted interview tool, artificial intelligence, child forensic interview, emotion recognition, forensic interviewing, vocal emotion analysis

## Abstract

Remote forensic interviews may restrict access to children’s nonverbal cues, potentially constraining affective assessment. This exploratory study examined whether children’s emotional states during forensic interviews could be identified using an artificial intelligence (AI)–assisted system that analyzes vocal emotional biomarkers, and whether AI-derived affective indices differ between an AI-assisted interview condition featuring real-time monitoring of children’s emotion and a traditional face-to-face interview condition. Fifty-nine children aged 4–8 years participated in simulated forensic interviews, yielding 2,084 utterances. Acoustic features were analyzed *post hoc* on recorded interview audio using a pretrained speech emotion model to estimate probabilities for happiness, anger, sadness, and neutral emotion. Regression analyses with participant-level clustering were conducted to account for the repeated-measures structure. Results indicated that anger probabilities, as well as anger-to-sadness and neutral-to-sadness ratios, were significantly higher in the AI-assisted condition. However, overall emotional distributions did not indicate increased distress associated with the AI-assisted modality, and dominant happiness did not differ significantly between groups. These findings suggest that AI-based vocal affect analysis may serve as a supplementary observational tool in forensic interviews while supporting the emotional validity of AI-assisted interview conditions. Particularly in contexts where visual cues are limited, AI-assisted approaches may offer a structured means of monitoring children’s affective changes without replacing professional judgment.

## 1 Introduction

Forensic interviews with children are a central investigative procedure in cases of suspected child abuse and sexual offenses (Karni-Visel et al., 2019). Interviewers are trained to elicit accurate and detailed accounts while minimizing retraumatization (Newlin et al., 2015). However, the interview context itself may induce psychological stress that disrupts memory retrieval and narrative production (Raffington et al., 2020; Vogel and Schwabe, 2016). Because children often hesitate to disclose abuse due to fear, shame, or anxiety (Hershkowitz et al., 2007), interviewers’ sensitivity to children’s emotional states is a critical determinant of interview quality. Children may become psychologically intimidated and experience emotional discomfort in face-to-face interview situations with authority figures, which can negatively affect accurate recall and hinder disclosure. Therefore, accurate assessment of children’s emotional states is particularly important in forensic interviews.

Contemporary protocols, including the revised NICHD model, emphasize rapport building and emotional responsiveness to facilitate disclosure (Hershkowitz and Lamb, 2020). Empirical evidence indicates that emotionally supportive interviewing practices reduce reluctance and enhance informational yield (Blasbalg et al., 2019), underscoring the close association between affective regulation and investigative outcomes.

The COVID-19 pandemic has highlighted the importance of maintaining evidence-based procedures in situations where traditional face-to-face interviews cannot be conducted (Brown et al., 2021; Vieth et al., 2020). The expansion of remote investigative practices has increased interest in AI-assisted interviews. In the present study, the AI-assisted condition involved a forensic interview supported by an AI tool designed to enable emotion monitoring and digital processing of interview data; however, emotional indices in this study were computed *post hoc* from recorded audio. The face-to-face condition involved a traditional in-person forensic interview without AI support. While AI-assisted formats offer logistical advantages, they may limit access to subtle nonverbal behavioral cues that are more readily available in face-to-face settings, raising concerns about the accurate interpretation of children’s emotional states (Mulay et al., 2021).

Previous research suggests that the quality of child forensic interviews depends less on interviewer intuition and more on the use of appropriate questioning techniques and structured procedures. This highlights the importance of approaches that promote consistency and objectivity in interviewing practices and suggests that AI-assisted systems may be useful in supporting standardized and systematic interview processes (Lyon, 2010).

Children participating in forensic interviews frequently exhibit emotional inhibition, particularly in cases involving intrafamilial allegations (Goodman-Brown et al., 2003). Inadequate recognition of affective distress may result in insufficient support and influence the quality and evaluation of statements (Golding et al., 2003; Stolzenberg et al., 2021). Conversely, appropriate acknowledgment of emotional states can attenuate stress and facilitate memory retrieval (Karni-Visel et al., 2019). Although basic emotion theories differ in their criteria and scope, happiness, sadness, and anger are consistently identified as core emotions across models (Ortony and Turner, 1990). Given their relevance in children’s emotional expression during forensic interviews, the present study focused on happiness, sadness, anger, and neutral states. Fear was excluded due to limitations in reliable measurement with the available acoustic features, and a neutral condition was included as a baseline (Tracy and Randles, 2011).

Given the challenges associated with accurately assessing children’s emotional states, one potential supplementary approach involves the analysis of vocal biomarkers. Acoustic properties of speech convey information regarding affective and psychophysiological states (Scherer, 1986), and advances in artificial intelligence enable computational estimation of emotional conditions from vocal features (Cummins et al., 2011). Such methods may be particularly relevant in system-assisted interviews, where visual information is constrained but audio data remain accessible.

Accordingly, the present study explores whether children’s emotional states during forensic interviews can be identified using an AI-assisted system that analyzes vocal emotional biomarkers. Specifically, this study examines whether AI-derived affective indices correspond to interview-related psychological processes and whether these indices differ between AI-assisted and face-to-face interview conditions. The AI-assisted interview system is conceptualized as a supplementary observational tool intended to support, rather than replace, professional judgment in forensic practice.

## 2 Methods

### 2.1 Participants

A total of 59 children participated, including preschool-aged children (4–5 years, *N* = 19) and school-aged children (6–8 years, *N* = 40). Emotional observations were derived from children’s vocal utterances produced during the interviews. A total of 2,084 utterances were analyzed, comprising 1,010 from the face-to-face interview group (*N* = 29) and 1,074 from the AI-assisted interview group (*N* = 30). Because multiple utterances were obtained from each participant, the data had a repeated-measures structure, which was accounted for in all analyses.

The mean age of the AI-assisted group was 6.03 years (SD = 1.45), with 18 girls (60.0%) and 12 boys (40.0%). The face-to-face group had a mean age of 6.48 years (SD = 1.50), with 18 girls (62.1%) and 11 boys (37.9%). *Mean T* scores (M ± SD) for the cognitive measures were as follows: Block Design (10.5 ± 3.28), Digit Span (11.2 ± 3.00), and Vocabulary (12.1 ± 3.18) in the AI-assisted group, and Block Design (10.9 ± 2.86), Digit Span (11.6 ± 2.49), and Vocabulary (11.8 ± 3.79) in the face-to-face group.

### 2.2 Emotion measurement

#### 2.2.1 Interview design and procedure

To approximate real-world investigative conditions, the study proceeded in three phases. First, children participated in a simulated disaster education session modeled after standardized safety training, including first-aid practice and an observed teacher conflict designed to induce mild negative affect. This procedure was designed to elicit ethically permissible levels of emotional response and was approved by the institutional review board (IRB) of Dongduk Women’s University (DDWU2501-01).

After a brief break, children were randomly assigned to either the AI-assisted or face-to-face interview condition using a color-based ball-drawing method. To control for cognitive differences, age-appropriate subtests of Wechsler Intelligence Scale were administered: younger children completed Block Design, Picture Memory, and Vocabulary from the Korean–Wechsler Preschool and Primary Scale of Intelligence–Fourth Edition (K-WPPSI-IV; Park et al., 2015), whereas older children completed Block Design, Digit Span, and Vocabulary from the Korean–Wechsler Intelligence Scale for Children–Fifth Edition (K-WISC-V; Kwak and Jang, 2019).

Children then underwent interviews according to their assigned condition. In the AI-assisted condition, interviews were conducted using an AI-based statement-support tool designed to enable automated emotion monitoring and digital processing of interview data; however, in the present study, emotional indices were computed *post hoc* from recorded audio rather than used in real time. In the face-to-face condition, children completed traditional in-person forensic interviews without AI support. All interviews followed the NICHD protocol (Lamb et al., 2007), beginning with rapport building and proceeding to questions about the disaster session and teacher conflict.

Audio and video were recorded with prior informed consent. Audio recordings were collected using a Galaxy Tab A9 + and stored in M4A format. Interviews were conducted in a counseling room approximately 2.5 meters wide and 3 meters long. To ensure consistent audio recording quality, a soundproof screen was installed on the desk.

#### 2.2.2 Data preparation

All vocal emotion analyses were conducted *post hoc* on recorded interview audio. Audio preprocessing was performed using Python-based open-source libraries. Specifically, voice activity detection (VAD) was conducted using Silero VAD to segment speech and silence regions. Acoustic feature extraction was carried out using librosa (for pitch, MFCC, mel-spectrogram, and spectral centroid) and openSMILE with the eGeMAPS feature set (for shimmer, harmonics-to-noise ratio, and loudness). All libraries are publicly available and widely used in the speech processing community.

Audio recordings were collected in environments in which interviewer and child speech could overlap. To isolate children’s speech, a preprocessing pipeline combining automatic speaker diarization and manual verification was applied. Initially, speaker segmentation and timestamp-based utterance segmentation were automatically performed. All segmented utterances were then manually reviewed to correct potential diarization errors.

Utterances identified as interviewer speech, non-child speech, semantically meaningless vocalizations (e.g., “ah,” “um”), or extended silence were excluded. In addition, utterances with excessive background noise, vocal distortion, or insufficient acoustic quality were removed based on predefined quality criteria. Only validated child utterances were retained for subsequent acoustic feature extraction and emotion probability estimation.

#### 2.2.3 Vocal feature extraction and emotion estimation

Emotion-related vocal biomarkers were extracted from finalized utterance-level audio recordings. Vocal biomarkers were operationalized as quantitative acoustic features independent of linguistic content that indirectly reflect internal affective states. To capture multiple signal-processing domains, features included prosodic indices (e.g., mean and variability of fundamental frequency [F0]), intensity-related measures, temporal characteristics (speech and pause duration), and spectral features derived from Mel-frequency cepstral coefficients (MFCCs) and Mel-spectrogram representations.

Extracted features were subsequently input into a pretrained speech emotion recognition (SER) model to estimate utterance-level emotion probabilities. The pretrained speech emotion recognition (SER) model was developed by the authors’ research team and is based on a machine learning architecture trained on a large-scale Korean speech corpus comprising more than 6,000 h of labeled audio data. The model had been trained on approximately 6,000 h of speech data across diverse age groups and generated continuous probability estimates for discrete emotional categories. In the present study, probabilities for happiness, anger, sadness, and neutral emotion were used as primary variables. Specifically, four independent SER models were developed, each dedicated to predicting one of the four emotion classes. For each audio segment, all four models were applied, and the highest scoring emotion output across the four models was selected and used as a feature. This value was then concatenated with the acoustic biomarker features to form the final feature vector.

The model was employed as a standardized measurement tool rather than for algorithm development or performance evaluation. Accordingly, the analysis focused on comparing emotional probability distributions across interview conditions.

### 2.3 Statistical analysis

To evaluate whether acoustic and emotion-related features could distinguish between interview conditions, classification was performed using a Random Forest classifier implemented in the scikit-learn library (Pedregosa et al., 2011) with default hyperparameters. Model performance was evaluated using repeated stratified k-fold cross-validation: a stratified 5-fold split (StratifiedKFold, shuffle = True) was repeated across 10 different random seeds, resulting in a total of 50 evaluation folds. This repeated scheme was adopted to reduce variance in performance estimates and improve the reliability of reported metrics.

To analyze group differences in utterance-level emotional data while accounting for the repeated-measures structure, regression analyses with cluster-robust standard errors were conducted, specifying participant identifiers as the clustering variable. This approach corrected for within-participant correlations across repeated observations.

Ordinary least squares (OLS) regression models were applied to continuous emotional probability outcomes, while binary logistic regression models with participant-level clustering were used to examine whether happiness emerged as the dominant emotion for each utterance. Separate models were estimated for composite positive and negative emotion indices as well as for individual emotion probabilities. To control for multiple testing, false discovery rate (FDR) correction was applied using the Benjamini–Hochberg procedure. Effect sizes were reported as regression coefficients for continuous outcomes and odds ratios for binary outcomes, along with 95% confidence intervals.

## 3 Results

### 3.1 Sample characteristics

The final analytical sample consisted of 59 participants, from whom a total of 2,084 utterance-level emotional observations were obtained (Table 1). Observations were approximately evenly distributed between the face-to-face interview group and the AI-assisted interview group with an average of approximately 35 utterances per participant. All statistical inferences were conducted with consideration of the repeated-measures structure, in which utterances were nested within participants.

**Table 1:** Sample characteristics.

	Face-to-face	AI-assisted	Total
*N*	29	30	59
Number of utterances	1,010	1,074	2,084
Utterances per child (mean)	34.8	35.8	35.3

### 3.2 Individual emotion scores and relative emotion ratios

Results of the analyses for individual emotions and relative emotion ratios based on sadness are presented in Table 2. In the individual emotion analyses, anger was significantly higher in the AI-assisted group than in the face-to-face group even after applying false discovery rate (FDR) correction (*B* = 3.302, *p*(FDR) = 0.003). In contrast, no statistically significant group differences were observed for the other individual emotions after FDR correction.

**Table 2:** Results for individual emotions and relative emotion ratios.

	Emotion	*β*	SE	*95%CI*	*p*	*p*(FDR)	Cohen’s *d*
Individual emotions	Angry	3.302	0.979	[1.383, 5.220]	0.001	0.003	0.087
	Happiness	3.721	2.838	[−1.841, 9.283]	0.190	0.190	0.39
	Neutral	2.796	1.269	[0.308, 5.283]	0.028	0.055	0.57
	Sad	−2.344	1.675	[−5.626, 0.938]	0.162	0.190	−0.18
Relative emotion ratios	Angry/Sad	0.112	0.044	[0.027, 0.198]	0.010	0.031	0.72
	Happiness/Sad	0.141	0.101	[−0.058, 0.339]	0.165	0.165	0.37
	Neutral/Sad	0.100	0.044	[0.014, 0.187]	0.023	0.035	0.52

In the relative emotion ratio analyses, both the anger-to-sadness ratio (*B* = 0.112, *p*(FDR) = 0.031) and the neutral-to-sadness ratio (*B* = 0.100, *p*(FDR) = 0.035) were significantly higher in the AI-assisted group compared to the face-to-face group. The happiness-to-sadness ratio did not differ significantly between groups.

### 3.3 Binary logistic regression analysis

To examine group differences in whether happiness emerged as the dominant emotion within each utterance, a logistic regression analysis with participant-level clustering was conducted. The results indicated that the odds of happiness being the most probable emotion were higher in the AI-assisted group than in the face-to-face group (*odds ratio* = 1.473); however, this effect was not statistically significant (95% *CI* [0.686, 3.163], *p* = 0.321).

In terms of observed probabilities, happiness was identified as the dominant emotion in 9.3% of utterances in the face-to-face group and 13.1% of utterances in the AI-assisted group (Table 3).

**Table 3:** Binary logistic regression results for dominant happiness.

Predictor	*Odds ratio*	95%CI	*p*	face to face probability	AI-assisted probability	Number of observation
AI-assisted: face to face	1.473	[0.686, 3.163]	0.321	0.093	0.131	2,084

## 4 Discussion

The present study did not aim to demonstrate the superiority of an AI-assisted interview system over trained interviewers’ professional judgment. Rather, acknowledging the structural constraints on observing nonverbal cues in remote forensic interview settings, the study examined whether voice-based emotional indicators could function as a complementary observational tool. Consistent with prior research (Hershkowitz and Lamb, 2020; Mulay et al., 2021), the AI-assisted interview system was conceptualized as decision support rather than as an automated authority (Sharma et al., 2023).

Importantly, the findings indicated no significant difference in emotional distress between AI-assisted and face-to-face interviews. Emotional distributions observed in the AI-assisted condition did not indicate heightened overall distress attributable to the interview modality. Instead, certain affective indicators—particularly anger-related probabilities—were more prominently detected. This does not necessarily imply increased emotional burden, but potentially enhanced sensitivity in capturing subtle vocal manifestations of tension, discomfort, or resistance that may be less perceptible in technology-mediated contexts (Doherty-Sneddon and McAuley, 2000; Rodríguez-Pellejero et al., 2024).

Anger is frequently conceptualized as an adaptive response to perceived threat or frustration and as a rapid signal of situational salience (Ekman, 1992). From this perspective, anger-related vocal cues should not be interpreted as negative outcomes per se, but as functional indicators of moments requiring heightened interviewer attunement or regulatory support. The observed differences in anger-to-sadness and neutral-to-sadness ratios further suggest that interview-condition effects may be more evident in relative emotional composition than in absolute emotional intensity. This interpretation aligns with theoretical models emphasizing affective configuration, including Russell’s circumplex model (Russell, 1980) and basic emotion theory (Ekman, 1992). Monitoring emotion ratios may therefore provide a nuanced method for detecting context-dependent affective shifts during investigative interviews (Wang et al., 2022).

Although binary logistic regression analyses did not yield statistically significant predictors, such findings should be interpreted cautiously. Naturalistic forensic speech data are inherently heterogeneous, characterized by variability in utterance length, acoustic quality, and nested observations within individuals (Durante et al., 2022; Deschamps-Berger et al., 2021). Given the modest sample size, wider confidence intervals reflect estimation uncertainty rather than definitive null effects (Altman and Bland, 1995).

The practical implication of this study lies in demonstrating that voice-based emotional analysis is not only feasible in forensic interview contexts but may also offer meaningful supplementary insight into children’s affective states. Because children often communicate distress indirectly through vocal modulation rather than explicit verbal disclosure (Kleye et al., 2022), systematic monitoring of vocal affect may enhance interviewers’ capacity to detect subtle emotional changes, particularly in remote settings where visual cues are attenuated. When shifts in anger-related indicators or emotional composition are observed, interviewers may adjust pacing, reformulate prompts, or provide emotional validation in a timely manner.

Several limitations warrant consideration. Variability in utterance quality and inherent measurement error in speech-based emotion estimation could not be fully controlled, and the sample size and contextual diversity were limited. Furthermore, the study did not directly assess how providing real-time emotional feedback influences interviewer decision-making. Future research should replicate these findings with larger and more heterogeneous samples and examine longitudinally how AI-assisted emotional monitoring affects interviewer behavior, interview dynamics, and investigative outcomes.

## Data Availability

The datasets generated for this study are not publicly available due to ethical restrictions related to research involving minor participants and the potential identifiability of voice data. Anonymized data may be made available from the corresponding author upon reasonable request and subject to institutional ethical approval. Requests to access the datasets should be directed to Jooyoung Lee, jylee7694@dongduk.ac.kr.
